# Intraoperative Application of Hyaluronic Acid in Achilles Tendon Repair: A Retrospective Cohort Study on Short-Term Functional Outcomes

**DOI:** 10.3390/medicina61101816

**Published:** 2025-10-10

**Authors:** Goker Yurdakul, Mehmet Okan Atahan, Aydogan Askin, Mehmet Fatih Uzun, Abdullah Iyigun, Fatih Golgelioglu, Haci Ali Olcar

**Affiliations:** 1 Department of Orthopedics and Traumatology, Faculty of Medicine, Yozgat Bozok University, 66100 Yozgat, Turkey; 2 Department of Orthopedics and Traumatology, Afyonkarahisar State Hospital, 03030 Afyonkarahisar, Turkey; 3 Department of Orthopedics and Traumatology, Antalya Training and Research Hospital, 07010 Antalya, Turkey; 4 Department of Orthopedics and Traumatology, Yozgat City Hospital, 66100 Yozgat, Turkey; 5 Department of Orthopedics and Traumatology, Umraniye Training and Research Hospital, 34764 Istanbul, Turkey

**Keywords:** achilles tendon, adhesion prevention, functional outcomes, hyaluronic acid, orthobiologics, tendon healing

## Abstract

*Background and Objectives*: This study aimed to investigate the effect of intraoperative hyaluronic acid (HA) application on short-term functional outcomes after open surgical repair of Achilles tendon rupture. *Materials and Methods*: This retrospective cohort study included 102 patients screened at three tertiary centers (January 2023–October 2024). After applying the exclusion criteria, 64 patients were analyzed (32 HA, 32 control), with group allocation based on the intraoperative use of HA. The primary outcomes were the American Orthopaedic Foot and Ankle Society (AOFAS) Score and the Achilles Tendon Total Rupture Score (ATRS), evaluated at 3, 6, and 12 months; the Short Form 36 (SF-36) Physical Component Summary (PCS) and Mental Component Summary (MCS) scores, assessed at 6 and 12 months; and physical-performance tests (heel-rise endurance, single-leg hop distance, and calf muscle strength), performed at 6 and 12 months. Complications, including re-rupture, wound infection, and delayed wound healing, were also recorded. *Results*: At 6 months, the HA group had higher AOFAS scores (mean difference: 9.0, 95% CI: 6.3–11.7, *p* = 0.008) and ATRSs (mean difference: 7.0, 95% CI: 3.8–10.2, *p* = 0.008) than the controls. The differences were smaller but remained significant at 12 months (AOFAS mean difference: 5.0, 95% CI: 2.5–7.5, *p* = 0.034; ATRS mean difference: 4.0, 95% CI: 1.1–6.9, *p* = 0.034). The SF-36 PCS and MCS scores were also superior in the HA group at 6 and 12 months (all *p* < 0.05). The physical-performance tests (heel-rise endurance, single-leg hop, calf strength) showed significant mean differences, with the HA group scoring higher at both timepoints (all *p* < 0.05). Complication rates were low and not significantly different between groups (all *p* > 0.05). *Conclusions*: Intraoperative HA application during open Achilles tendon repair was associated with improvements in short-term functional recovery, general health status, and physical performance without increasing complication rates. These findings support the use of HA as a safe adjunct to optimize tendon gliding; however, prospective randomized controlled trials are needed to confirm these results.

## 1. Introduction

Achilles tendon rupture is one of the most common tendon injuries of the lower extremities, particularly affecting physically active individuals between the ages of 30 and 50 [1]. These injuries are usually initiated during rapid dorsiflexion or plantarflexion with strong resistance, frequently occurring during sporting activities. Even with the increasing advancements in diagnostic methods and surgical techniques, Achilles tendon rupture remains a clinical problem due to the long rehabilitation period, lasting functional impairment, and risk of re-rupture. Therefore, optimizing treatment approaches and enhancing postoperative recovery continue to be important objectives in orthopedic practice [2]. In addition, dysmetabolic diseases such as diabetes mellitus, hyperlipidemia, and metabolic syndrome have been shown to negatively affect tendon homeostasis by impairing collagen cross-linking, increasing oxidative stress, and reducing tendon vascularity, which may predispose patients to rupture and delayed healing. Therefore, recognition and optimal management of these comorbidities play a key role in treatment planning and prognosis [3,4,5]. Currently, minimally invasive and open surgical methods are the preferred approaches for Achilles tendon repair [6,7], and are supported by the I.S.Mu.L.T. Achilles tendon rupture guidelines, which recommend individualized treatment planning based on patient age, activity level, and comorbidities [8]. Although minimally invasive methods are claimed to have lower rates of wound-related complications, some reports indicate that such methods increase the risk of sural nerve injury compared to open surgical methods [7,8,9,10,11]. Therefore, open surgical repair continues to be widely preferred, particularly to minimize the risk of nerve damage.

In recent years, a variety of orthobiologic agents—such as platelet-rich plasma, autologous conditioned serum, and mesenchymal stem cells—have been investigated as adjuncts in the treatment of Achilles tendon ruptures, primarily aiming to enhance tendon regeneration and biomechanical strength [12,13]. Although these agents have shown potential in increasing collagen synthesis and altering tendon histology, most have primarily been concerned with enhancing intrinsic tendon healing [14]. Nonetheless, excessive scar formation and postoperative adhesions between the tendon and its adjacent structures, primarily the paratenon, have been noted as key functions limiting functional recovery. Tendon adhesions can restrict gliding motion, lead to stiffness, and restrict total ankle mobility, all of which affect the transition from pre-injury activity levels [15,16]. Therefore, besides biological healing, the prevention of peritendinous adhesion formation is also important for optimizing postoperative outcomes.

Hyaluronic acid (HA) is a naturally occurring glycosaminoglycan with a broad distribution in connective, epithelial, and neural tissues, best known for its viscoelasticity and lubrication properties. Furthermore, it is essential for maintaining tissue hydration, mediating inflammation, and facilitating cell migration [17,18,19]. HA is presently being used in orthopedic and surgical environments to reduce friction between tissues and limit adhesion formation, especially in tendon and joint surgeries [20,21,22]. Many experimental animal studies have demonstrated that the intraoperative use of HA can decrease the extent of peritendinous adhesions and ultimately facilitate the gliding of the repaired tendon [23,24]. Because the anti-adhesive and anti-inflammatory effects of HA are most pronounced during the early phases of tendon healing, short-term outcomes were prioritized in the present study to capture its potential clinical benefit in the critical early healing window [25,26]. While this provides promising preclinical evidence, there is limited clinical evidence on the efficacy of HA in improving function in human patients with Achilles tendon rupture. However, to our knowledge, clinical studies specifically investigating the intraoperative application of HA in Achilles tendon ruptures remain limited. This represents an important gap in the current orthopedic literature that warrants further clinical investigations.

This study aims to investigate the effect of intraoperative HA application between the tendon and paratenon on short-term postoperative functional outcomes in patients undergoing open surgical treatment for Achilles tendon rupture.

## 2. Materials and Methods

### 2.1. Study Design

This retrospective cohort study was conducted at three tertiary care centers. Ethical approval was obtained from the Yozgat Bozok University Institutional Ethics Committee (Approval Number: 2025-GOKAEK-251-285; date: 8 January 2025) prior to initiating data collection. Written informed consent was obtained from all participants, and the study was conducted in accordance with the ethical standards of the Declaration of Helsinki.

### 2.2. Patient Selection

This retrospective cohort study included patients who underwent open surgical repair for complete acute Achilles tendon rupture between January 2023 and October 2024 at three tertiary care centers. All surgical procedures were performed by a total of four orthopedic surgeons specialized in Achilles tendon surgery, each with at least 5 years of clinical experience and performing approximately 10–15 Achilles tendon repairs annually.

**Figure 1:**
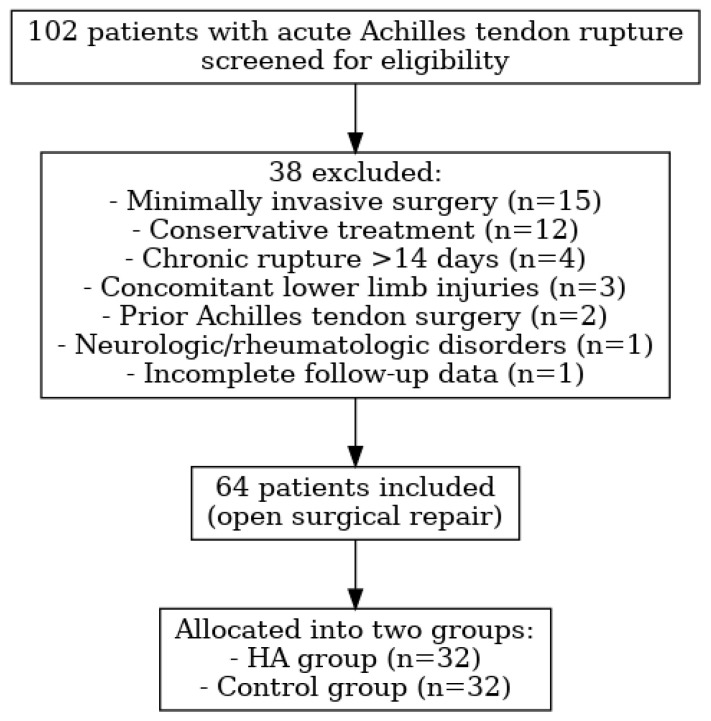
Flow diagram of patient selection, exclusion, and allocation into study groups.

Initially, 102 patients treated for acute Achilles tendon rupture were screened for eligibility. To maintain methodological consistency and reduce variability, 38 patients were excluded based on the following criteria: they had previously undergone minimally invasive surgery (n = 15), conservative treatment (n = 12), or Achilles tendon surgery (n = 2); presented with chronic rupture > 14 days (n = 4), concomitant lower limb injuries (n = 3), or neurologic or rheumatologic disorders (n = 1); or had incomplete follow-up data (n = 1). Consequently, 64 patients were included in the final analysis. Patients were recruited consecutively from three tertiary centers between January 2023 and October 2024 to minimize selection bias. A flowchart of the patient selection process is illustrated in Figure 1.

The included patients were aged between 18 and 60 years, presented with unilateral complete acute Achilles tendon rupture within 14 days of injury, and had undergone primary open repair. Patients were assigned to one of two groups based on the intraoperative preference of surgeons regarding the application of HA. Surgeons who routinely utilized HA intraoperatively formed the HA group (n = 32), whereas surgeons who did not apply HA constituted the control group (n = 32). The decision to apply HA was entirely based on individual surgeons’ clinical routines and preferences, independent of patient demographics, injury severity, or temporal factors. All included cases had at least 12 months of clinical follow-up and were managed using a standardized postoperative rehabilitation protocol.

The following demographic and clinical data were recorded: age, gender, body mass index (BMI), side of injury (right or left), limb dominance, systemic comorbidities (including diabetes mellitus, hypertension, coronary artery disease, and chronic respiratory diseases), smoking status (current smoker or non-smoker), time interval from injury to surgery, and duration of postoperative follow-up.

### 2.3. Surgical Technique

All patients underwent open surgical repair under spinal or general anesthesia in the prone position, with a pneumatic tourniquet applied to the proximal thigh. A posteromedial longitudinal incision measuring approximately 8–10 cm was made over the Achilles tendon. After identifying the rupture location, debridement of devitalized tendon tissue was performed to identify healthy tendon ends. The repair was made using the Krackow suture technique with number 2 non-absorbable braided polyester sutures (Ethibond^®^, Ethicon Inc., Somerville, NJ, USA), which achieved stable fixation and restored continuity of the tendon. This repair was followed by closure of the paratenon; this was carried out with 4-0 absorbable polydioxanone sutures to conserve the original tendon sheath and minimize scar formation, which is supported by previous findings indicating the paratenon’s crucial role in tendon healing [27].

In the treatment group, after tendon repair and before paratenon closure, high-molecular-weight HA gel (Dreamvisc^®^ 32 mg/1.6 mL; approximate molecular weight: 3000 kDa; VSI Biotechnology GmbH, Leinfelden-Echterdingen, Germany) was applied intraoperatively in a single dose using the spreading technique between the repaired tendon and the overlying paratenon. In the control group, no anti-adhesive agent was used. In both groups, the subcutaneous tissue and skin were closed in anatomical layers.

### 2.4. Postoperative Follow-Up and Rehabilitation

After surgery, all patients were immobilized in a below-knee cast with the ankle positioned with approximately 20° of plantarflexion for the first two weeks. This was followed by the use of a controlled ankle motion boot, allowing gradual dorsiflexion and partial weight-bearing until the sixth week. Full weight-bearing was permitted thereafter based on clinical tolerance. Prophylactic low-molecular-weight heparin was administered once daily for 14 days to prevent venous thromboembolism. All patients received a standard postoperative regimen including oral antibiotics for 5–7 days and acetaminophen or nonsteroidal anti-inflammatory drugs for pain control as needed. Physiotherapy was initiated in the sixth week, beginning with range-of-motion exercises and progressing to strengthening and proprioceptive training. Return to sports was typically allowed between the 6th and 9th postoperative months, depending on individual recovery.

### 2.5. Functional Outcome Evaluation

Functional outcomes were evaluated using two validated scoring systems widely applied in foot and ankle surgery: the American Orthopaedic Foot and Ankle Society (AOFAS) Ankle–Hindfoot Score and the Achilles Tendon Total Rupture Score (ATRS). Both scores were assessed at 3, 6, and 12 months postoperatively. Evaluations were conducted by an independent orthopedic specialist who was not involved in the index surgery and thus remained unaware of group allocation at the time of outcome assessment. Although this is a retrospective study and formal patient blinding was not feasible, this approach minimized measurement bias. The Turkish validity and reliability of the ATRS were established by Kaya et al. [28], and the AOFAS score adaptation used in this study was previously reported by Analay et al. [29], with higher scores on both scales reflecting better functional status and fewer symptoms. In addition, general health status and physical function were assessed using the 36-Item Short Form Health Survey (SF-36), which includes both the Physical Component Summary (PCS) and the Mental Component Summary (MCS), with evaluations performed 6 and 12 months postoperatively. Higher scores in each domain reflect better health status and physical functionality. The Turkish validity and reliability of SF-36 were established by Kaya et al. [30] in 2018.

To assess objective functional recovery, physical-performance tests were conducted preoperatively and repeated at the 6th and 12th postoperative months. These tests included heel-rise endurance, single-leg hopping distance, and calf muscle strength measurements. Heel-rise endurance (seconds) was assessed by counting how many continuous repetitions patients could perform until fatigue. Single-leg hopping distance (cm) was evaluated by instructing patients to hop as far as possible horizontally from a standing position, measuring the distance from the take-off line to the back of the heel upon landing. Calf muscle strength was quantified using a handheld dynamometer (MicroFET2, Hoggan Health Industries, West Jordan, UT, USA), measuring maximal isometric plantarflexion force in Newtons (N). These tests aimed to objectively compare the recovery of both muscle strength and functional capacity between the HA and control groups.

### 2.6. Statistical Analysis

All analyses were performed in IBM SPSS Statistics (v30, IBM Corp., Armonk, NY, USA). Continuous variables are presented as mean ± standard deviation (SD) or median as appropriate, while categorical variables are presented as n (%). Normality was assessed with the Shapiro–Wilk test. Between-group comparisons used the independent-samples *t*-test for normally distributed variables and the Mann–Whitney U test otherwise. Categorical variables were compared with the χ^2^ test or Fisher’s exact test as appropriate.

For primary outcomes (AOFAS, ATRS, and SF-36 domain scores at 6 months), we report effect sizes as mean differences with 95% confidence intervals (CIs) and *p*-values. For complications, we report risk differences with 95% CIs. Missing data constituted < 5% of observations and were handled by listwise deletion, with no imputation performed. Baseline covariates with potential confounding impact (age, BMI, smoking status, diabetes, and other comorbidities) were compared between groups to assess balance.

A two-tailed α value of 0.05 was considered statistically significant. Given a single prespecified primary timepoint (6 months), no multiplicity adjustment was applied to the primary outcomes; secondary and subgroup analyses are considered exploratory. Post hoc power analysis (two-sample comparison, α = 0.05, two-sided) indicated that the study had >80% power to detect a between-group difference of ≥7.0 AOFAS points with n = 32 per group, based on the observed pooled SD ≈ 10 points in our cohort.

## 3. Results

A total of 64 patients were included in the study, with 32 in the HA group and 32 in the control group. The baseline demographic and clinical characteristics of both groups are summarized in Table 1.

**Figure 2:**
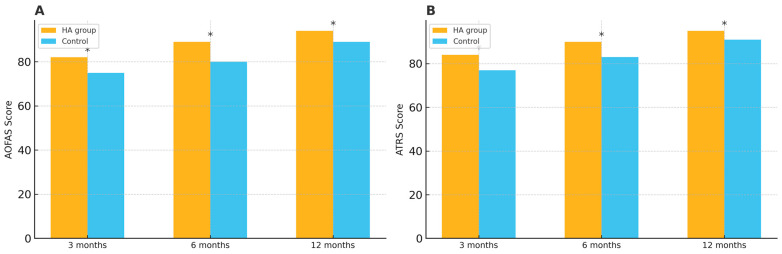
(**A**) American Orthopedic Foot and Ankle Society (AOFAS) scores at 3, 6, and 12 months. (**B**) Achilles Tendon Total Rupture Scores (ATRSs) at 3, 6, and 12 months. * indicates *p* < 0.05 between groups.

In terms of functional recovery, the HA group demonstrated significantly higher AOFAS scores and ATRSs at the 3rd and 6th months compared to the control group. At 6 months, the mean difference was 9.0 points for AOFAS (95% CI: 6.3–11.7, *p* = 0.008) and 7.0 points for ATRS (95% CI: 3.8–10.2, *p* = 0.008). Although the difference were slightly smaller at 12 months, the HA group maintained better functional outcomes (AOFAS mean difference: 5.0, 95% CI: 2.5–7.5, *p* = 0.034; ATRS mean difference: 4.0, 95% CI: 1.1–6.9, *p* = 0.034) (Table 2). The differences in AOFAS scores and ATRSs between the groups at each follow-up are illustrated in Figure 2.

**Figure 3:**
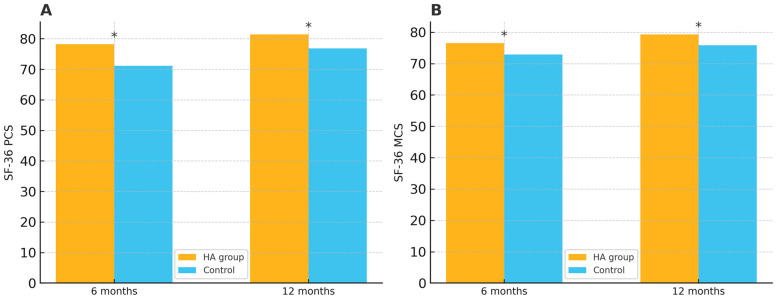
(**A**) Short Form 36 (SF-36) Physical Component Summary (PCS) scores at 6 and 12 months. (**B**) SF-36 Mental Component Summary (MCS) scores at 6 and 12 months. * indicates *p* < 0.05 between groups.

Additionally, both the Physical Component Summary (PCS) and the Mental Component Summary (MCS) scores of the SF-36 were significantly higher in the HA group at 6 and 12 months. At 6 months, the mean difference was 7.1 points for PCS (95% CI: 4.3–9.9, *p* = 0.013) and 3.6 points for MCS (95% CI: 0.6–6.6, *p* = 0.019). At 12 months, the HA group continued to achieve superior scores, with mean differences of 4.6 (95% CI: 1.8–7.4, *p* = 0.027) for PCS and 3.4 (95% CI: 0.4–6.4, *p* = 0.031) for MCS (Table 3). The differences in SF-36 PCS and MCS scores between the groups at 6 and 12 months are illustrated in Figure 3.

**Figure 4:**
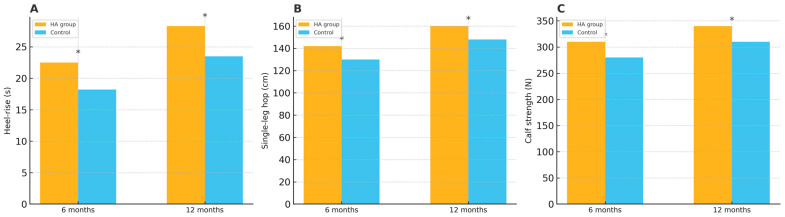
(**A**) Heel-rise endurance at 6 and 12 months. (**B**) Single-leg hopping distance at 6 and 12 months. (**C**) Calf muscle strength at 6 and 12 months. * indicates *p* < 0.05 between groups.

The evaluation of physical performance at 6 and 12 months showed that the HA group achieved significantly better results in heel-rise endurance, single-leg hopping distance, and calf muscle strength. At 6 months, the mean differences were 5.3 s for heel-rise endurance (95% CI: 3.1–7.5, *p* = 0.015), 8.2 cm for single-leg hopping (95% CI: 4.8–11.6, *p* = 0.018), and 7.8 N for calf muscle strength (95% CI: 3.7–11.9, *p* = 0.012). At 12 months, the HA group maintained superior performance, with mean differences of 4.7 s (95% CI: 2.4–7.0, *p* = 0.022), 6.5 cm (95% CI: 3.2–9.8, *p* = 0.027), and 8.9 N (95% CI: 4.8–13.0, *p* = 0.021), respectively (Table 4). The heel-rise endurance, single-leg hopping distance, and calf muscle strength results are illustrated in Figure 4.

During the 12-month follow-up, complication rates were low and comparable between groups. The risk difference for re-rupture was 0.0% (95% CI: −9.3% to 9.3, *p* = 1.000), and wound infection occurred in one patient (3.1%) in the HA group and two patients (6.3%) in the control group (risk difference: −3.2%, 95% CI: −14.6% to 8.2, *p* = 0.554). Delayed wound healing was observed in two patients (6.3%) in the HA group and one patient (3.1%) in the control group (risk difference: +3.2%, 95% CI: −8.2% to 14.6, *p* = 0.554). No cases of deep vein thrombosis were recorded in either group (Table 5).

## 4. Discussion

The most important finding of the current study was that the intraoperative application of HA during open Achilles tendon repair resulted in superior functional outcomes, as demonstrated by significantly higher AOFAS scores and ATRSs in the early postoperative period, as well as improved SF-36 scores and physical-performance test results throughout the 12-month follow-up. These potential positive results are likely due to the anti-adhesive and lubricating properties of HA, which probably decreased the risk of postoperative adhesions developing between the tendon and paratenon, thereby preserving the gliding of the tendon and encouraging early functional recovery.

Previous studies have demonstrated that demographic characteristics such as age, BMI, and smoking behavior can negatively impact tendon healing and post-operative outcomes. Noback et al. [31] found that increased BMI was associated with an increased risk of a Achilles tendon rupture. Chen et al. [32] further identified both elevated BMI and smoking status as independent risk factors for Achilles tendon rupture, noting that smoking may impair tendon healing by reducing vascularity and collagen synthesis. In our study, the proportion of smokers was similar between groups, thereby minimizing the potential confounding effect of smoking on our results.

Our analysis indicated that the functional benefit observed in the HA group at 3 and 6 months postoperatively was reduced at 12 months. This observation may indicate that HA’s anti-adhesive effects are most beneficial during early healing of a tendon, presumably due to the reduction in adhesions surrounding the tendon and increased gliding of the tendon through the sheath. As the healing process and natural remodeling occur over time, we cannot rule out the idea that these declines are a biological function; therefore, the differences at 12 months may be reflective of the biological process of healing. This finding coincides with previous studies reporting that the greatest benefits of HA occurred in the earliest part of recovery [33,34].

In line with this finding, previous experimental studies have shown that adhesions typically develop in the first few weeks following injury, a time when the inflammatory response is at its peak and tissues are proliferating, which may be crucial for the long-term function of a tendon [16,20]. Animal studies also emphasize that treatments intended to reduce adhesion formation are most effective when performed in the early postoperative period, highlighting the importance of early clinical interventions [23,25].

Another important finding of our study was that at 6 and 12 months postoperatively, intraoperative HA application improved not only tendon-specific functional outcomes, but also general health status (SF-36 PCS and MCS scores) and physical performance. These results suggest that HA’s role in reducing tendon adhesions may positively impact overall health perceptions and physical performance. Similar beneficial effects of HA have been reported in studies involving flexor and patellar tendons, highlighting its potential in enhancing tendon healing and early functional recovery [21,25].

This finding also suggests that peritendinous adhesions may not only compromise tendon-specific functional outcomes, but also negatively affect overall physical performance and general health status. Supporting this concept, previous studies have shown that tendon adhesions can significantly limit physical function, impair mobility, and decrease patients’ perceived quality of life. For example, Wong et al. [16] demonstrated that adhesions in flexor tendons directly limited hand function and quality of life. Similarly, Yao et al. [15] reported that tendon adhesions adversely impacted joint mobility and overall functional recovery. Therefore, strategies aimed at reducing adhesion formation could potentially improve broader aspects of patient well-being beyond local tendon function alone.

In the present study, complication rates, including re-rupture, wound infection, delayed wound healing, and deep vein thrombosis, were comparable between the HA and control groups. Both groups demonstrated identical re-rupture rates, aligning closely with previously reported rates following open Achilles tendon repair [2]. This finding suggests that intraoperative HA application does not compromise the structural integrity of the repair, as the risk of re-rupture was not increased. Wound-related complications occurred at low frequencies and without significant differences between groups, indicating that HA does not adversely affect wound healing or infection risk. Moreover, no cases of deep vein thrombosis were recorded, further supporting the safety of HA as an adjunct in open Achilles tendon repair [21].

High-molecular-weight HA (approximately 1800 kDa) was selected for its advantageous biological effects on tendon healing, which have been extensively documented in the recent literature. Several preclinical and clinical studies highlight the efficacy of high-molecular-weight HA preparations, which exhibit enhanced viscoelasticity, lubrication properties, and the capacity to maintain tissue hydration, thereby reducing tendon friction and adhesion formation [35]. Additionally, laboratory findings by Osti et al. [36] demonstrated that high-molecular-weight HA significantly increases tendon-derived cell viability and proliferation, promotes collagen type I synthesis, and positively influences extracellular matrix remodeling. Clinical evidence further supports the use of ultrasound-guided high-molecular-weight HA injections in Achilles and patellar tendinopathies, showing significant clinical improvements, reduced pain and inflammation, and decreased neovascularization [37]. Based on these comprehensive preclinical and clinical findings, the choice of high-molecular-weight HA in our study to achieve the expected optimal biological and clinical outcomes in tendon healing was justified.

In the present study, we utilized non-absorbable braided polyester sutures (Ethibond^®^) for tendon repair, primarily due to their superior biomechanical strength, stability, and widespread clinical acceptance in Achilles tendon surgery. Non-absorbable sutures provide robust mechanical fixation, reducing the likelihood of early mechanical failure and re-rupture, thus ensuring safer and more reliable postoperative rehabilitation [38,39]. Although concerns have been raised in the literature regarding the potential risk of increased infection or delayed wound healing associated with non-absorbable sutures, recent evidence suggests that there are no significant differences in infection rates, reactions to foreign bodies, or rerupture risk compared to absorbable sutures in Achilles tendon repair [40]. Our postoperative complication rates remained consistent with those reported in previous studies [40,41], indicating that meticulous surgical techniques and careful postoperative management effectively minimized these risks.

In tendon repair, biomaterials such as platelet-rich plasma (PRP), mesenchymal stem cell (MSC)-seeded scaffolds, and barrier membranes have been explored to enhance healing and reduce adhesions. PRP delivers growth factors that may accelerate collagen synthesis and modulate inflammation, though clinical trial results are inconsistent [12]. MSC-based scaffolds provide cellular support and sustained release of trophic factors but are technically complex and less practical in routine settings [42]. Barrier membranes and hydrogel films (including hyaluronan derivatives) act as physical separators to limit peritendinous adhesion formation [43,44]. However, the intraoperative use of hyaluronic acid has several advantages over these methods: it is simple, is biocompatible, requires no elaborate equipment, and has shown favorable safety and functional outcomes in short- to medium-term follow-ups.

A major strength of the current study is that, to the best of our knowledge, it is the first clinical evaluation specifically examining the efficacy of intraoperative HA application during Achilles tendon rupture repair. Additionally, our study employed multiple validated outcome measures, including tendon-specific functional scores (AOFAS score and ATRS), general health status (SF-36), and objective physical-performance tests, thus providing a comprehensive assessment of recovery. Another important strength of this study is that the baseline demographic and clinical characteristics, including age, gender, BMI, smoking status, comorbidities, limb dominance, and side of injury, were carefully matched between groups, minimizing potential confounding bias and enhancing the reliability of our comparative results, further strengthening the validity and reliability of our comparative findings. However, these results are primarily applicable to the general adult population with acute Achilles tendon rupture and may not fully reflect outcomes in elite athletes or patients with atypical activity levels.

This study has several limitations in accordance with STROBE recommendations. First, its retrospective cohort design carries an inherent risk of selection and information bias, although we attempted to minimize this by consecutively screening all eligible patients across three tertiary centers and using standardized inclusion and exclusion criteria. Second, while the outcome assessor was blinded, surgeon and patient blinding were not possible, which may have introduced performance bias. Surgeon variability could also have influenced the functional outcomes despite adherence to a standardized surgical technique. Third, although baseline characteristics were comparable between groups, and major confounders such as age, BMI, smoking status, and comorbidities were balanced, residual confounding cannot be entirely excluded. Fourth, the sample size was moderate, and post hoc power analysis indicated that the study had >80% power to detect a ≥7-point difference in AOFAS scores, but it may have lacked the power to detect smaller differences or rare complications. Finally, the follow-up period was limited to 12 months, and thus, long-term outcomes such as tendon re-rupture beyond the first year and late-onset complications were not assessed. This limitation may limit the generalizability of our findings to long-term scenarios.

Future investigations should focus on large-scale, multicenter, randomized controlled trials to validate our findings and minimize potential selection bias. Direct assessment of peritendinous adhesions using advanced imaging modalities, such as magnetic resonance imaging or intraoperative contrast-enhanced techniques, as well as quantitative gliding resistance measurements, would provide a more objective understanding of the correlation between HA application and improved tendon gliding. Furthermore, studies exploring optimal HA formulations, molecular weights, and repeated dosing regimens are warranted to determine the most effective therapeutic protocol. Complementary biomechanical and histological studies in animal models may help elucidate the biological mechanisms through which HA enhances tendon healing, ultimately contributing to the development of standardized clinical guidelines for its use in Achilles tendon repair.

## 5. Conclusions

The present study demonstrates that intraoperative application of HA during open surgical repair of Achilles tendon ruptures leads to significant improvements in tendon-specific functional outcomes, general health status, and physical performance, particularly in the short-term postoperative period. These favorable effects are most likely due to HA’s anti-adhesive properties, which reduce peritendinous adhesion formation and facilitate optimal tendon gliding. Given the safety profile and beneficial clinical outcomes demonstrated, intraoperative HA application can be considered a valuable adjunctive approach in Achilles tendon surgery. However, larger prospective randomized controlled studies with longer follow-up periods, as well as biomechanical analyses using animal models, are warranted to further confirm these findings and establish clearer long-term clinical recommendations.

## Figures and Tables

**Table 1:** Baseline demographic and clinical characteristics of the study groups.

Parameters	HA Group (n = 32)	Control Group (n = 32)	*p*-Value
Mean age (years)	39.2 ± 6.1	40.1 ± 5.8	0.645
Gender (Male, %)	22 (68.8%)	21 (65.6%)	0.790
BMI (kg/m^2^)	27.4 ± 3.2	27.8 ± 3.1	0.712
Side of injury, right (n, %)	17 (53.1%)	18 (56.3%)	0.802
Limb dominance, right (n, %)	26 (81.3%)	25 (78.1%)	0.756
Smoking status (n, %)	7 (21.9%)	8 (25.0%)	0.767
Time interval from injury to surgery (days)	4.2 ± 1.6	4.4 ± 1.7	0.632
Comorbidities (n, %)	6 (18.8%)	6 (18.8%)	1.000
Diabetes mellitus (n, %)	3 (9.4%)	3 (9.4%)	1.000
Hypertension (n, %)	2 (6.3%)	2 (6.3%)	1.000
Cardiovascular disease (n, %)	1 (3.1%)	0 (0%)	0.313
Chronic respiratory disease (n, %)	0 (0%)	1 (3.1%)	0.313

HA: hyaluronic acid; BMI: body mass index. Data are presented as numbers (%) or means ± standard deviation.

**Table 2:** Functional outcome scores at 3, 6, and 12 months postoperatively.

Measure	Time	HA (Mean ± SD)	Control (Mean ± SD)	Mean Diff (95% CI)	*p*-Value
AOFAS score	3 months	82 ± 6.1	75 ± 5.8	7.0 (4.0–10.0)	0.021
	6 months	89 ± 5.3	80 ± 5.5	9.0 (6.3–11.7)	0.008
	12 months	94 ± 4.7	89 ± 5.1	5.0 (2.5–7.5)	0.034
ATRS	3 months	84 ± 7.2	77 ± 6.9	7.0 (3.5–10.5)	0.021
	6 months	90 ± 6.5	83 ± 6.1	7.0 (3.8–10.2)	0.008
	12 months	95 ± 5.9	91 ± 5.6	4.0 (1.1–6.9)	0.034

AOFAS: American Orthopedic Foot and Ankle Society; ATRS: Achilles Tendon Total Rupture Score; HA: hyaluronic acid. Data are presented as means ± SD and mean differences with 95% confidence intervals.

**Table 3:** Short Form 36 (SF-36) Physical and Mental Component Summary scores at 6 and 12 months postoperatively.

Measure	Time	HA (Mean ± SD)	Control (Mean ± SD)	Mean Difference (95% CI)	*p*-Value
SF-36 PCS	6 months	78.2 ± 4.8	71.1 ± 5.4	7.1 (4.3–9.9)	0.013
	12 months	81.4 ± 5.0	76.8 ± 5.5	4.6 (1.8–7.4)	0.027
SF-36 MCS	6 months	76.5 ± 5.1	72.9 ± 5.3	3.6 (0.6–6.6)	0.019
	12 months	79.3 ± 5.2	75.9 ± 5.4	3.4 (0.4–6.4)	0.031

SF-36: Short Form 36; PCS: Physical Component Summary; MCS: Mental Component Summary; HA: Hyaluronic acid; CI: Confidental interval; Data presented as mean ± SD and mean difference with 95% confidence interval.

**Table 4:** Physical-performance test results at 6 and 12 months postoperatively.

Measure	Time	HA (Mean ± SD)	Control (Mean ± SD)	Mean Difference (95% CI)	*p*-Value
Heel-rise Endurance (s)	6 months	35.4 ± 4.2	30.1 ± 3.9	5.3 (3.1–7.5)	0.015
	12 months	41.2 ± 5.1	36.5 ± 4.6	4.7 (2.4–7.0)	0.022
Single-leg Hopping (cm)	6 months	78.5 ± 6.8	70.3 ± 5.9	8.2 (4.8–11.6)	0.018
	12 months	85.7 ± 7.3	79.2 ± 6.4	6.5 (3.2–9.8)	0.027
Calf Muscle Strength (N)	6 months	120.5 ± 8.3	112.7 ± 7.9	7.8 (3.7–11.9)	0.012
	12 months	127.8 ± 8.5	118.9 ± 8.1	8.9 (4.8–13.0)	0.021

HA: Hyaluronic acid; s, seconds; cm, centimeters; N: Newtons; sd: standard deviation; CI: confidental interval. Data are presented as means ± SD and mean differences with 95% confidence intervals.

**Table 5:** Complication rates during the 12-month follow-up period.

Complication	HA (n, %)	Control (n, %)	Risk Difference (95% CI)	*p*-Value
Re-rupture	1 (3.1%)	1 (3.1%)	0.0% (−9.3% to 9.3%)	1.000
Wound infection	1 (3.1%)	2 (6.3%)	−3.2% (−14.6% to 8.2%)	0.554
Deep vein thrombosis	0 (0%)	0 (0%)	0.0% (NA)	1.000
Delayed wound healing	2 (6.3%)	1 (3.1%)	+3.2% (−8.2% to 14.6%)	0.554

HA: hyaluronic acid; CI: confidence interval; NA: not applicable. Data are presented as numbers (%) or mean differences with 95% confidence intervals.

## Data Availability

The data can be obtained from the corresponding author upon request.

## Abbreviations

The following abbreviations are used in this manuscript:

ATRS	Achilles Tendon Total Rupture Score
BMI	Body mass index
HA	Hyaluronic acid
MCS	Mental Component Summary
PCS	Physical Component Summary
SF-36	Short Form 36
